# Ceftaroline-Associated Encephalopathy: A Rare Adverse Effect

**DOI:** 10.7759/cureus.14795

**Published:** 2021-05-01

**Authors:** Aswin Srinivasan, Blake Bennie, Krina Viroliya, Ramesh Kesavan, Siva T Sarva

**Affiliations:** 1 Internal Medicine, HCA Houston Kingwood/University of Houston College of Medicine, Kingwood, USA; 2 Department of Pharmacy - Internal Medicine, HCA Houston Kingwood, Kingwood, USA; 3 Pulmonary and Critical Care - Internal Medicine, HCA Houston Kingwood/University of Houston College of Medicine, Kingwood, USA

**Keywords:** ceftaroline, encephalopathy

## Abstract

In this case report, we present a patient who suffered ceftaroline-associated encephalopathy, while receiving ceftaroline for acute bacterial skin and skin structure infections (aBSSSIs) that resolved after cessation of the suspected agent. We recommend close monitoring for encephalopathy in patients with creatinine clearance (CrCl) of <50 mL/min receiving ceftaroline.

## Introduction

Ceftaroline is a novel cephalosporin that was approved in 2010 by the Food and Drug Administration (FDA) [1]. Unique to beta lactams, ceftaroline has activity against methicillin-resistant Staphylococcus aureus (MRSA). It was therefore studied in acute bacterial skin and skin structure infections (aBSSSIs) and community acquired pneumonia (CAP) in the CANVAS and FOCUS randomized trials, respectively. According to the CANVAS trial, ceftaroline is well-tolerated overall as only 3% of patients in the trial reported adverse effects such as nausea, headache, diarrhea, and pruritus. None of these trials mentioned altered mental status or encephalopathy as an adverse effect of ceftaroline [2-5]. In this case report, we report a patient who developed encephalopathy after administration of ceftaroline.

## Case presentation

A 76-year-old female with past medical history of hypertension, diabetes mellitus, heart failure with preserved ejection fraction (HFpEF), and chronic obstructive pulmonary disease (COPD) on home oxygen, presented with worsening shortness of breath, lower extremity edema, cough, and one episode of diarrhea. Additionally, she had pain and redness in the inner aspect of her right arm from a previous intravenous site, suggestive of cellulitis. At the emergency department (ED), chest radiograph showed cardiomegaly and vascular congestion. Vitals at admission include: temperature of 36.7 C, blood pressure of 168/97, pulse of 86 bpm, respiration rate of 19 rpm, and O2 saturation of 98% using nasal cannula at 2 L/min. Labs include serum sodium of 130 mEq/L, potassium of 4.4 mEq/L, blood urea nitrogen (BUN) of 38, serum creatinine of 1.1, and pro-BNP of 2920. Transthoracic echocardiography (TTE) revealed an ejection fraction of 60-64% with normal wall motion, systolic function, and indeterminate diastolic function.

For right arm cellulitis, IV cefazolin was initiated for a total of four days. On hospital day 3, wound and blood cultures were positive for MRSA and cefazolin was changed to IV vancomycin. Secondary seeding workup was done with transesophageal echocardiography (TEE), which showed no evidence of cavitations, thrombus, or vegetations. Bacteremia was persistent on vancomycin therefore therapy was switched to daptomycin on day 9. Her blood cultures remained positive on daptomycin, and ceftaroline was added on day 15. On day 16, the arterial blood gas (ABG) showed pH of 7.41, PCO2 of 51.5 mm Hg, PO2 of 86.5 mm Hg, HCO3- of 32.3 mm Hg, O2 sat of 96.4 (Table 1). The serum creatinine was 1.3 on day 14, the day prior to the start of ceftaroline. The creatinine increased to 1.6 on day 17. On hospital day 16-17, she became progressively confused. CT head/brain without contrast showed no intracranial hemorrhage or mass effect. MRI brain could not be obtained. Thyroid stimulating hormone (TSH), vitamin B12, and ammonia levels were within normal limits. Ceftaroline was discontinued. On hospital day 20, the patient became alert, awake and obtundation improved. On day 19, blood culture resulted in no growth of MRSA. The patient was eventually discharged with a PICC line for a six-week course of daptomycin.

**Table 1:** Arterial blood gas of the patient during hospital course. Partial Pressure of CO2 = mm Hg, Partial Pressure of O2 = mm Hg, O2 saturation = %, Bicarbonate = mEq/L.

	Day 16	Day 18	Day 19	Day 20
pH	7.41	7.41	7.35	7.39
PCO2	51.5	48.6	52.1	54.0
PO2	86.5	54.6	131.6	82.3
O2 saturation	96.4	87.8	98.8	95.7
Bicarbonate	32.3	31.0	28.9	32.6

## Discussion

Ceftaroline is a well-tolerated antibiotic whose side effect profile includes rash, diarrhea, constipation, nausea, elevated transaminases, and electrolyte disturbances such as hypokalemia. Even though encephalopathy is a well-known complication of other beta-lactam antibiotics, to date, only one study has reported ceftaroline-associated encephalopathy [6, 7]. In this study of 28 patients with estimated glomerular filtration rates (eGFR) < 30 mL/min who received ceftaroline for ≥5 days, three patients developed encephalopathy. Our patient developed encephalopathy with eGFR of 42 mL/min and creatinine clearance (CrCl) of 33 mL/min. Using the Naranjo scale to assess causality, the likelihood of ceftaroline-induced encephalopathy is ‘probable’ for this patient [8]. Even though electroencephalogram (EEG) findings of ceftaroline does not exist in literature, triphasic waves have been associated with cefepime, a closely related beta-lactam [9]. Home medications were reconciled and other confounding causes of encephalopathy were ruled out. Additionally, ABGs were analyzed to rule out hypercapnic encephalopathy as this patient has the history of COPD. Hypercapnia is unlikely to be the cause, as the degree of encephalopathy in this patient is not explained by the degree of elevation in CO2. Furthermore, the trend of the PCO2 was not consistent with the trend of encephalopathy. One limitation of this study includes lack of a confirmatory lumbar puncture. The patient did not have nuchal rigidity, focal weakness or any seizure like activity on exam. The symptoms promptly resolved with stopping of the medications without any neurological interventions making a primary neurological cause for encephalopathy unlikely.

Despite its only labeled indications being for aBSSSIs and CAP, ceftaroline has been used off-label for other MRSA infections, including infective endocarditis (IE). Ceftaroline has been linked to improved sterilization of blood when used with daptomycin in patients with MRSA IE versus daptomycin or vancomycin monotherapy [10]. Doses used in these case series are often higher than those studied in CANVAS/FOCUS trials, however, with sparse data on the safety and tolerability of increased dosing. Dosing of ceftaroline in CANVAS/FOCUS trials exclusively used the 600 mg q12h regimen, with only a reduction to 400 mg q12h for those patients with a CrCl of 30-50 mL/min. Our patient received the appropriate dosage adjustment as listed in the package insert [1], but nevertheless suffered an adverse event that resulted in stopping ceftaroline therapy. No cases of encephalopathy have been reported with total dosage of <800 mg/day.

## Conclusions

Beta-lactam associated encephalopathy is a well-established phenomenon, especially among those with concomitant renal failure. We present a case of a patient who suffered ceftaroline-associated encephalopathy, while receiving ceftaroline for MRSA that resolved after cessation of the suspected agent. We recommend close monitoring for encephalopathy in patients with CrCl of <50 mL/min receiving ceftaroline for any indication.
